# Intravenous immunoglobulin for postpolio syndrome: a systematic review and meta-analysis

**DOI:** 10.1186/s12883-015-0301-9

**Published:** 2015-03-22

**Authors:** Yao-Hsien Huang, Hung-Chou Chen, Kuang-Wei Huang, Po-Chih Chen, Chaur-Jong Hu, Chin-Piao Tsai, Ka-Wai Tam, Yi-Chun Kuan

**Affiliations:** Department of Neurology, Taipei Medical University-Shuang Ho Hospital, New Taipei City, Taiwan; Center for Evidence-Based Health Care, Taipei Medical University-Shuang Ho Hospital, New Taipei City, Taiwan; Department of Physical Medicine and Rehabilitation, Taipei Medical University-Shuang Ho Hospital, New Taipei City, Taiwan; Department of Gastroenterology, College of Medicine, Taipei Medical University, Taipei, Taiwan; Faculty of Medicine, School of Medicine, National Yang-Ming University, Taipei, Taiwan; Department of Gastroenterology, Taipei Medical University-Shuang Ho Hospital, New Taipei City, Taiwan; College of Medical Science and Technology, Taipei Medical University, Taipei, Taiwan; Department of Neurology, School of Medicine, Taipei Medical University, Taipei, Taiwan; Department of Neurology, Neurological Institute, Taipei Veterans General Hospital, Taipei, Taiwan; Department of Surgery, School of Medicine, College of Medicine, Taipei Medical University, Taipei, Taiwan; Graduate Institute of Clinical Medicine, College of Medicine, Taipei Medical University, Taipei, Taiwan; Division of General Surgery, Department of Surgery, Taipei Medical University-Shuang Ho Hospital, Taipei, Taiwan; Center for Evidence-Based Medicine, College of Medicine, Taipei Medical University, Taipei, Taiwan; Taipei Medical University-Shuang Ho Hospital, 291 Zhongzheng Road, Zhonghe District, New Taipei City, 23561 Taiwan

**Keywords:** Post-polio syndrome, Intravenous immunoglobulin, Fatigue, Meta-analysis, Muscle strength, Quality of life

## Abstract

**Background:**

Postpolio syndrome (PPS) is characterized by progressive disabilities that develop decades after prior paralytic poliomyelitis. Because chronic inflammation may be the process underlying the development of PPS, immunomodulatory management, such as intravenous immunoglobulin (IVIg) administration, may be beneficial.

**Methods:**

We performed a systematic review and meta-analysis of published randomized controlled trials (RCTs) and prospective studies that evaluated the efficacy of IVIg in managing PPS. Electronic databases, including PubMed, EMBASE, CINAHL, and the Cochrane Central Register of Controlled Trials, were searched for articles on PPS published before December 2014. The primary outcomes were pain severity, fatigue scores, and muscle strength. The secondary outcomes were physical performance, quality of life (QoL), and cytokine expression levels.

**Results:**

We identified 3 RCTs involving 241 patients and 5 prospective studies involving 267 patients. The meta-analysis of pain severity (weighted mean difference [WMD] = −1.02, 95% confidence interval [CI] = −2.51 to 0.47), fatigue scores (WMD = 0.28, 95% CI −0.56 to 1.12), and muscle strength revealed no significant differences between the IVIg and the placebo group. Regarding QoL, the RCTs yielded controversial outcomes, with improvement in only certain domains of the Short Form 36 (SF-36). Moreover, one prospective study reported significant improvement on SF-36, particularly in patients aged younger than 65 years, those with paresis of the lower limbs, and high pain intensity.

**Conclusion:**

The present review indicated that IVIg is unlikely to produce significant improvements in pain, fatigue, or muscle strength. Thus, routinely administering IVIg to patients with PPS is not recommended based on RCTs. However, a potential effect in younger patients with lower limbs weakness and intense pain requires confirmation from further well-structured trials.

## Background

Postpolio syndrome (PPS) may develop from prior paralytic poliomyelitis after an interval (usually ≥ 15 years) of stable neurological function. It is characterized by a gradual onset of persistent and progressive, new or further muscle weakness or decreased endurance. Muscle atrophy, pain, and fatigue are common, whereas hypoventilation, dysphagia, or dysphonia may develop as well [1]. The March of Dimes International Conference on PPS in 2000 proposed additional diagnostic criteria for PPS, stating that the symptoms should persist for at least 1 year, and other causes should be excluded [2,3]. The World Health Organization estimated that approximately 20 million people worldwide have sequelae of poliomyelitis [3], mostly from the epidemics in the 1940s and 1950s in Western countries. A previous study reported the prevalence of PPS to be 15% to 80% in patients with previous paralytic polio on the basis of different clinical diagnostic criteria [4]. Although poliomyelitis has been almost eradicated worldwide through the widespread use of polio vaccines, PPS will continue to remain a concern in developing countries for subsequent decades.

To date, no curative or specific treatment exists for PPS. Potential management approaches include pharmacological (modafinil, intravenous immunoglobulin [IVIg], pyridostigmine, lamotrigine, amantadine, high-dose prednisone, coenzyme Q10, selegiline, insulin-like growth factor, and human growth hormone) and nonpharmacological interventions (muscle strengthening, rehabilitation, and static magnetic fields). However, the evidence available from randomized controlled studies is inadequate for formulating a definite conclusion regarding the alleviation of activity limitations, fatigue, and pain as well as the improvement of muscle strength [3].

The most widely accepted pathogenesis for the muscle-related effects of PPS is uncompensated reinnervation through collateral sprouting that increases the motor unit areas in response to denervation from prior acute poliomyelitis. Thus, progressive muscle weakness occurs years after prior acute poliomyelitis. The cause of the ongoing denervation remains unclear and is probably multifactorial, including factors such as stress or the overuse of motor units, normal aging, the persistence of poliovirus fragments, a patient’s genetic background, and chronic inflammation [2]. In addition, a previous study reported increased concentrations of several cytokines in the cerebrospinal fluid (CSF) of patients with PPS [5]. In consideration of the involvement of a possible immune-mediated process in the development of PPS, IVIg, a polyclonal antibody preparation collected from the plasma pools of healthy blood donors [6], may confer beneficial effects against PPS, as observed in other neuroinflammatory conditions [7]. To date, although several studies have investigated the effects of IVIg in PPS treatment [5,8-14], these studies reported inconclusive findings, most likely because of the inadequate evidence levels and small sample sizes. Therefore, we conducted a systematic literature review and meta-analysis of randomized controlled trials (RCTs) and prospective trials to evaluate the effectiveness of IVIg in PPS treatment.

## Methods

### Selection criteria

We reviewed RCTs or prospective trials that evaluated the outcomes of IVIg in PPS treatment. For inclusion in our study, the trials were required to describe the following: (1) inclusion and exclusion criteria used for patient selection, (2) IVIg dosing strategies, and (3) evaluations of clinical outcomes. We excluded trials that met as least one of the following criteria: (1) the clinical outcomes stated were unclear or (2) duplicate reporting of patient cohorts.

### Search strategy and study selection

The studies were identified by performing keyword searches of electronic databases, namely PubMed, EMBASE, CINAHL, the Physiotherapy Evidence Database (PEDro), SCOPUS, the Cochrane Central Register of Controlled Trials, and the ClinicalTrials.gov registry (http://clinicaltrials.gov/). The following terms and Boolean operator were used in MeSH and free-text searches: *postpolio syndrome* OR *poliomyelitis* OR *postpoliomyelitis, intravenous immunoglobulin* OR *IVIg*. The “related articles” facility in PubMed was used to broaden the search. No language restrictions were applied. The final search was performed in December 2014. We attempted to identify additional studies by searching the reference sections of relevant papers and contacting known experts in the field. The systematic review described here was accepted by the online PROSPERO international prospective register of systematic reviews of the National Institute for Health Research (CRD42014013305).

### Data extraction

Yi-Chun Kuan and Yao-Hsien Huang independently extracted details on the RCTs and prospective trials regarding the participants, inclusion and exclusion criteria, IVIg dosing strategy, outcome parameters, and complications.

The individually recorded decisions of both reviewers were compared, and any disagreements were resolved according to the evaluation of a third reviewer, Ka-Wai Tam.

### Methodological quality appraisal

Yi-Chun Kuan and Yao-Hsien Huang independently appraised the methodological quality of each RCT based on the “risk of bias” method recommended by the Cochrane Collaboration [15]. Several domains were assessed: allocation generation; allocation concealment; blinding of participants, personnel, and outcome assessors; completeness of outcome data; freedom from selective reporting; and freedom from other biases.

### Outcome assessments

The primary outcomes of our study consisted of the evaluation of the efficacy of IVIg treatment 2 to 3 months after IVIg administration according to the severity of pain and fatigue, and improvement of muscle strength. The secondary outcomes were physical performance, quality of life (QoL), and changes in cytokine expression at 1, 2, and 12 months. The pain intensity was assessed using visual analog scales (VASs) [16]. The fatigue severity was investigated using the Fatigue Severity Scale (FSS) and the Multidimensional Fatigue Index [17]. QoL was evaluated using the Short Form 36 (SF-36) [18]. Muscle strength was measured using the Medical Research Council (MRC) grading scale and a dynamometer [19]. The Physical Activity Scale for the Elderly (PASE) [20], 6-minute walk test (6MWT) [21], and time up and go test (TUG) [22] were conducted to evaluate physical performance. Inflammatory cytokines including tumor necrosis factor α (TNF-α), transforming growth factor β, interferon (IFN-γ and IFN-β), and interleukins (IL-1β, IL-4, IL-6, IL-10, IL-13, and IL-23) in peripheral blood mononuclear cells (PBMCs) and CSF cells were measured using an enzyme-linked immunosorbent assay [12] or real-time polymerase chain reaction to determine the anti-inflammatory effects of IVIg treatment [5,13].

### Statistical analysis

Statistical analysis was conducted using the Review Manager (Version 5.3) computer software (Cochrane Collaboration, Oxford, England). The meta-analysis of RCTs was conducted according to the Preferred Reporting Items for Systematic Reviews and Meta-analysis guidelines [23]. When necessary, standard deviations were estimated according to the reported confidence interval (CI) limits, standard error, or range values [24]. The mean difference was calculated for continuous outcomes, and the weighted mean difference (WMD) was analyzed. The precision of an effect size was reported as a 95% CI. A pooled estimate of the mean difference was calculated using the DerSimonian and Laird random-effect model [25]. The data were pooled only for studies that exhibited adequate clinical and methodological similarities. Statistical heterogeneity was assessed using the *I*^*2*^ test, with *I*^*2*^ quantifying the proportion of total outcome variability that was attributable to the variability among the studies.

## Results

### Characteristics of the trials

**Figure 1:**
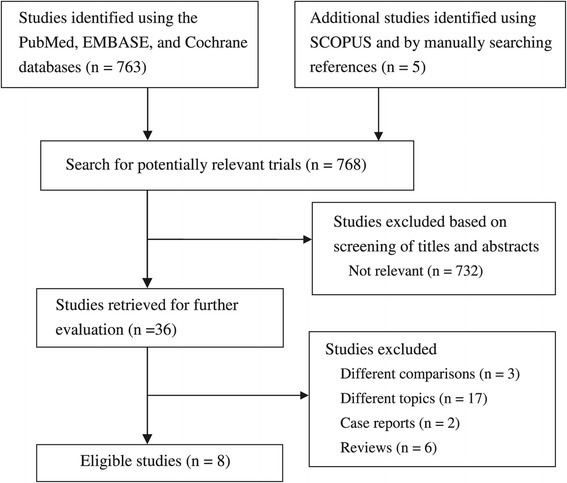
**Flowchart of the stuy selection process.**

The flow chart in Figure 1 shows the screening and selection processes of the trials. Our initial search yielded 768 studies, of which 732 were deemed ineligible by screening their titles and abstracts. The remaining 28 reports were excluded from our final analysis for the following reasons: 6 were review articles, 2 were case reports, 3 used different comparisons, and 17 discussed different topics. The remaining 8 eligible trials were included in our analysis [5,8-14]; of these, 3 were RCTs and 5 were prospective trials.

Table 1 shows the characteristics of the eligible studies. The 8 trials had been published between 2004 and 2013, with sample sizes ranging from 14 to 142 patients. All of the patients had been diagnosed with PPS, with ages ranging from 36 to 88 years. Four trials included patients with established PPS who fulfilled the diagnostic criteria proposed by Halstead et al. [26,27], 2 trials used the March of Dimes criteria [28], and one trial used the European Federation of Neurological Societies guidelines [4]; in addition, these studies used various IVIg dosing strategies: 90 g in 3 days [5,8-11,13], 2 g/kg infused for 2 to 4 days [12], or 0.4 g/kg/day for 5 days [14], respectively. In all of these studies, several clinical parameters were assessed at the baseline. The follow-up period ranged between 1 to 6 months in most studies, except one study that monitored pain severity for up to 1 year after treatment [5]; this study was a clinical extension study from a previous double-blind placebo controlled trial [10] of 135 patients with PPS. Of the 3 RCTs included in our review, Bertolasi et al. defined QoL limited to physical component score (PCS) of SF-36 as the primary outcomes. Farbu et al. studied changes in pain, fatigue and muscle strength 3 months after intervention. Whereas the Scandinavian group assessed strength in the clinically polio-affected muscle and QoL measured with the SF-36 PCS as their primary outcomes [10,12,14]. Secondary outcomes included evaluation of the effects of IVIg treatment on gait [5,10,14], physical activity [8,10,11], balance and sleep quality [10], and fatigue [10,12,14] as well as changes in cytokine expression levels in PBMCs and CSF cells, before and after IVIg treatment [5,12,13]. Among all studies, the clinical outcomes were evaluated using various assessment tools including questionnaires; for example, the SF-36 was used to evaluate QoL, the VAS was used to evaluate pain, and the FSS was used to evaluate fatigue. The other measurement tools were a dynamometer, which was used to assess muscle strength, and simple clinical tests such as the TUG, 6MWT, and MRC, which were used to evaluate gait, physical activity, and muscle strength.

**Table 1:** **Characteristics of the selected trials**

**Study**	**Study design**	**Inclusion criteria**	**Number of patients (% men)**	**Age (years)**	**Intervention**	**Outcomes**
Bertolasi [2013] [14]	RCT	18-70 y, history of paralytic polio; clinical stability > 15 years; new symptoms fulfilling the EFNS guidelines criteria	I: 24 (50) P: 26 (50)	I: 54.9 ± 5.7 P: 58.3 ± 5.6	I: 0.4 g/kg/d × 5 d P: saline at the same volume	QoL, gait, muscle strength, fatigue, pain at 2 and 4 mo
Farbu [2007] [12]	RCT	Ambulatory patients fulfilling the diagnostic criteria for PPS	I: 10 (40) P: 10 (30)	I: 59.9 ± 6.2 P: 58.7 ± 6.8	I: 2 g/kg infused for 2–4 d P: saline at the same volume	Pain, muscle strength, fatigue at 1, 3, and 6 mo; changes in serum and CSF cytokine levels at 1 mo
Gonzalez [2006] [10]	RCT	18-75 y, history of polio; increased weakness, fatigue, and pain fulfilling the diagnostic criteria for PPS	I: 73 (29) P: 69 (42)	I: 61.5 ± 9.2 P: 59.0 ± 10.0	I: 30 g × 3 d, twice at 3-mo intervals P: glucose water at the same volume	QoL, balance, fatigue, gait, muscle strength, physical activity, sleep quality, and pain at 9 − 13wk
Gonzalez [2012] [5]	Prospective	18-75 y, history of polio; increased weakness, fatigue, and pain fulfilling the diagnostic criteria for PPS	I: 20 (30) P: 21 (43)	I: 61.7 (52–75)^†^ P: 61.9 (46–75)	I: 30 g × 3 d, twice at 3-mo intervals P: glucose water at the same volume	Cytokine expression in PBMCs and CSF, QoL, gait, pain at 1 year
Gonzalez [2004] [13]	Prospective	Patients fulfilling the diagnostic criteria for PPS	I: 16 (56)	I: 58.5 (43–70)^†^	I: 30 g × 3 d	Cytokine expression in PBMCs and CSF at 6–8 wk
Kaponides [2006] [8]	Prospective	Patients fulfilling the diagnostic criteria for PPS	I: 14 (57)	I: 57 (43–67)^†^	I: 30 g × 3 d	QoL at 2 and 6 mo; physical performance and muscle strength at 2 mo
Östlund [2012] [11]	Prospective	Patients fulfilling the diagnostic criteria for PPS	I: 113 (45)	I: 66 ± 10	I: 90 g infused for 3 d	QoL, physical activity, and pain at 6 mo
Werhagen [2011] [9]	Prospective	Patients with PPS	I: 45 (36)	I: 61 (36–88)^†^	I: 30 g × 3 d	Pain at 6 mo

CSF, cerebrospinal fluid; EFNS, European Federation of Neurological Societies; I: intravenous immunoglobulin group; IVIg, intravenous immunoglobulin; P, placebo group; PBMCs, peripheral blood mononuclear cells; Polio, poliomyelitis; PPS, post-polio syndrome; QoL, quality of life; Data presented as the mean ± standard deviation except ^†^mean (range).

Table 2 presents the methodological quality of the 3 RCTs. Two studies reported acceptable methods of randomization and described the methods of allocation concealment [10,12]. All studies reported patient blinding as well as the outcome assessors used [10,12,14], and one trial reported the blinding of caregivers [10]. Two studies used an intention-to-treat analysis [12,14]. For all studies, the acceptable number of patients lost to follow-up was < 20%. None of the studies reported any relevant information that could be used to determine the risk of bias attributable to incomplete data. Most studies reported a small sample size as the primary limitation of their study. Other biases included the following: (a) one RCT reported a marked disparity in the baseline muscle strength measurements between the intervention and the placebo groups [12]; (b) one RCT assessed PPS-affected muscle strength as a primary outcome, and the secondary outcomes included changes in the muscle strength measurements of 3 arbitrarily defined muscle groups as unaffected muscles. This arbitrariness might have caused muscle groups that were already assessed for primary outcomes to be included in evaluation of secondary outcomes, potentially altering accuracy of the results [10].

**Table 2:** **Assessment of the methodological quality of the randomized controlled trials**

**Study [year]**	**Country**	**Allocation generation**	**Allocation concealment**	**Blinding**	**Data analysis**	**Loss to follow-up**	**Selective reporting**	**Other bias**
Bertolasi [2013] [14]	Italy	Unclear	Unclear	Blinded patients and assessors	ITT	0	Low risk	Low risk
Farbu [2007] [12]	Norway	Notes drawn	Adequate	Blinded patients and assessors	ITT	0	Low risk	Intergroup differences in baseline assessments of muscle strength
Gonzalez [2006] [10]	Sweden	Computer generated	Adequate	Blinded caregivers, patients, and assessors	PP	4.9%	Low risk	Affected muscles were assessed for both primary and secondary outcomes

The risk of bias was assessed according to the methods recommended by the Cochrane Collaboration.

ITT, Intention-to-treat; PP, Per-protocol.

### Pain

**Figure 2:**
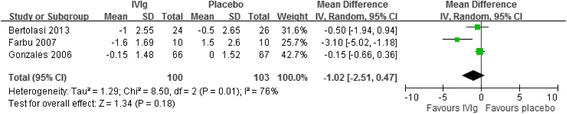
**Forest plot of IVIg treatment compared with a placebo.** Outcome: Changes of visual analog pain scale, cm.

Pain was assessed using a 10-cm VAS (0 = no pain, 10 = worst possible pain). We compared the outcome measures during the initial 2 to 3 months following IVIg treatment. The pooled mean difference in the pain scores of the 3 included RCTs was −1.02 (95% CI: −2.51 to 0.47), and no significant differences were observed in the pain scores between the treatment and the control groups [10,12,14] (Figure 2). The *I*^*2*^ value was 76%, indicating heterogeneity among the studies.

Three prospective studies that evaluated pain according to a VAS reported significant benefits of IVIg after 3, 6, or 12 months of treatment [5,9,11] (Table 3). In addition, 2 prospective studies reported a significant reduction of bodily pain (BP), a subdomain of the physical component score (PCS) of the SF-36 [8,11], particularly in patients aged younger than 65 years and those with paresis of the lower limbs and a VAS pain intensity higher than 2 cm [11].

**Table 3:** **Outcomes before and after intravenous immunoglobulin treatment of patients with postpolio syndrome**

**Study**	**Assessment**	**Results**
Bertolasi [2013] [14]	SF-36, 6MWT, muscle strength, FSS, and VAS at 2 and 4 mo	Significant improvements in RP, mental component score, and RE of the SF-36 at 2 mo.
Farbu [2007] [12]	VAS, muscle strength, and FSS at 1, 3, and 6 mo; TNF-α, IFN-γ, IL-6, IL-1β, IFN-β, and IL-10 levels in CSF and serum measured using enzyme-linked immunosorbent assay at 1 mo	Significant alleviation of in pain at 3 mo; significantly decreased CSF TNF-α levels (*P* = 0.028; no significant differences after regression because of the differences in the baseline values).
Gonzalez [2006] [10]	SF-36, VAS, MFI-20, PASE, 6MWT, TUG, muscle strength, balance, and sleep quality at 9–13 wk	Significant improvements in the strength of the affected muscles and the vitality scores of the SF-36 and PASE.
Gonzalez [2012] [5]	SF-36, 6MWT, and VAS; IFN-γ, TNF, IL-10, IL-13, IL-23, and TGF-β levels in PBMCs and CSF at 1 year	Significant improvements in the physical components of the SF-36, VAS scores, and 6MWT. Significant decrease in the CSF IFN-γ and IL-23 levels but significant increase in the CSF IL-13 levels.
Gonzalez [2004] [13]	IFN-γ, TNF-α, IL-10, and IL-4 levels in PBMCs and CSF at 6–8 wk	Significant decrease in the CSF IFN-γ and TNF-α mRNA levels and significant increase in the PBMCs IL-4 levels.
Kaponides [2006] [8]	SF-36 at 2 and 6 mo; 6MWT and muscle strength at 2 mo	Significant improvements in the PF, RP, BP, GH, VT, SF, and MH subdomains of the SF-36 at 2 and 6 mo.
Östlund [2012] [11]	SF-36, PASE, and VAS at 6 mo	Significant improvement of pain in patients with VAS score > 2 cm, age < 65 y, and paresis of the lower limbs. Significant improvements in the BP, VT, SF, RE subdomains of the SF-36.
Werhagen[2011] [9]	VAS at 6 mo	31/45 (69%) patients exhibited significant improvements in the mean VAS scores, which decreased from 53 to 42 (*p* = 0.001).

6MWT, 6-minute walk test; BP, bodily pain; CSF, cerebrospinal fluid; FSS, fatigue severity scale; GH, general health; IL, interleukin; IFN, interferon; MFI, multidimensional fatigue index; MH, mental health; PASE, physical activity scale for the elderly; PBMCs, peripheral blood mononuclear cells; PF, physical functioning; QoL, quality of life; RE, role emotional; RP, role physical; SF, social functioning; TGF: transforming growth factor; TNF, tumor necrosis factor; TUG, time up and go test; VAS, visual analog scale; and VT, vitality.

### Fatigue

**Figure 3:**
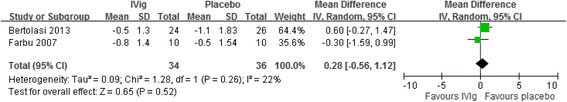
**Forest plot of IVIg treatment compared with a placebo.** Outcome: Changes of Fatigue Severity Scale.

Two of the 3 RCTs that reported reduction of fatigue scores 2 and 3 months after treatment, respectively [12,14], were subjected to meta-analysis (Figure 3). No significant changes were observed between the IVIg and the placebo groups (WMD = 0.28; 95% CI −0.56 to 1.12), and no heterogeneity was evident (*I*^*2*^ = 22%).

### Muscle strength

**Figure 4:**
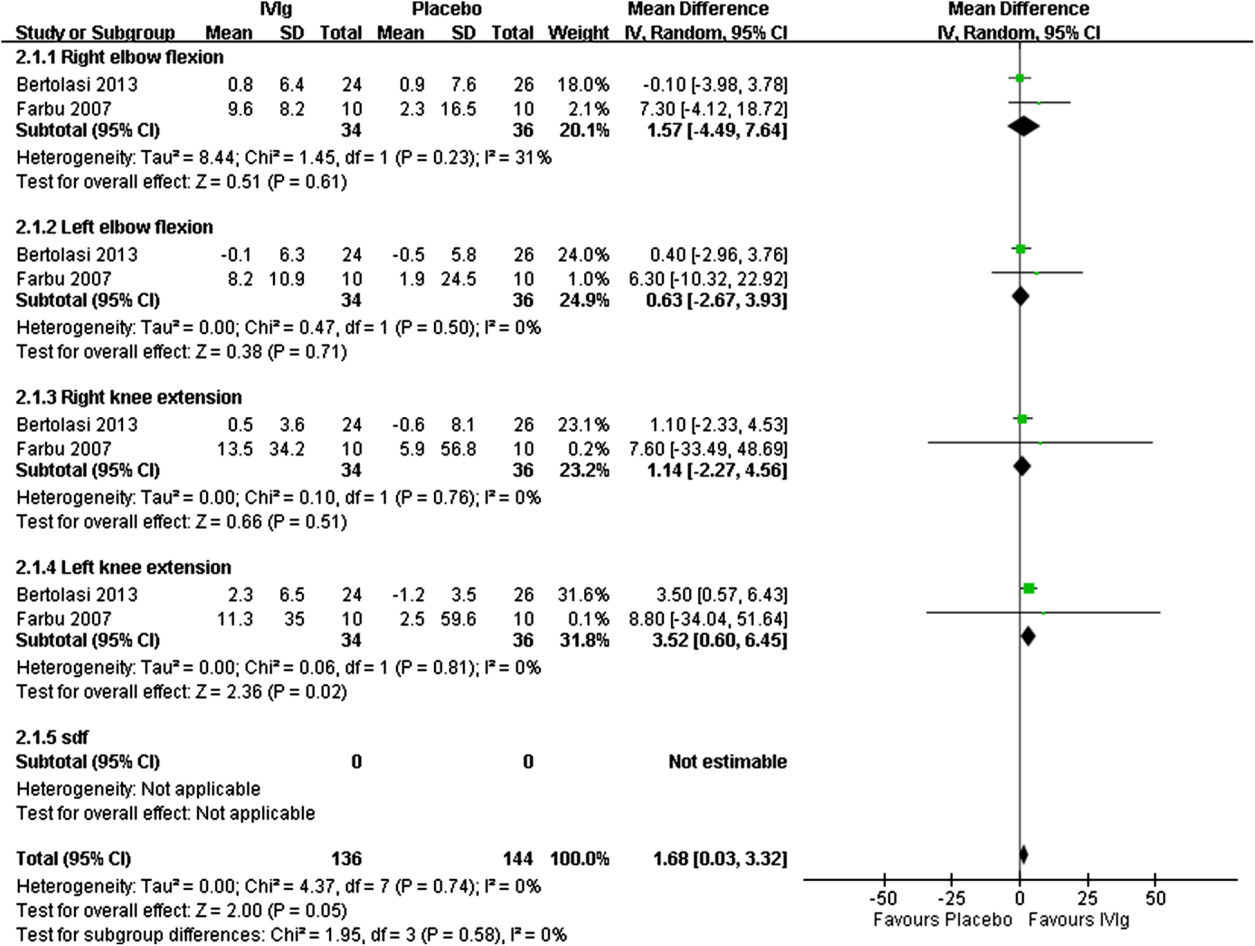
**Forest plot of IVIg treatment compared with a placebo.** Outcome: Changes of muscle strength, Nm.

Muscle strength was measured using a dynamometer in the 3 RCTs [10,12,14]. Data from 2 RCTs were pooled for analysis, and no significant effect on the muscle strength of four limbs was found after IVIg (Figure 4) [12,14]. Gonzalez et al. reported median strength of polio-affected muscles difference between IVIg and placebo groups of 8.6% in favor of the IVIg group (*P* = 0.029) [10]. However, we did not include this data in our analysis because this study differs with the other RCTs in that the muscles selected for assessment were symptomatic or not. In addition, only one prospective study evaluated muscle strength and revealed no significant improvement after IVIg treatment [8].

### Physical performance

To evaluate physical performance, 2 RCTs and 2 prospective studies used the 6-MWT, whereas one RCT and one prospective study used PASE. One RCT reported significant improvements in PASE results [10], and only one prospective study reported improvements in 6-MWT results [5].

### Quality of life

The PCS, including the physical functioning, role physical, BP, and perception of general health scores, and mental component score (MCS), comprising the vitality (VT), social functioning (SF), role limitations because of emotional problems (RE), and mental health scores, of the SF-36, were assessed in 2 RCTs [10,14] and 3 prospective studies [5,8,11]. Data from the 2 RCTs were not pooled for analysis because one RCT did not report the mean and standard deviation scores essential for pooling data [14]. No significant improvement in the overall PCS was reported in the 2 RCTs. However, Bertolasi et al. reported that IVIg treatment significantly benefitted the overall MCS and RE subdomain score [14]. In addition, Gonzalez et al. reported a significant increase in VT [10].

Among the eligible prospective studies, Gonzales et al. [5] and Kaponides et al. [8] observed significant improvements in the overall PCS 1 year and 2 to 6 months after IVIg treatment, respectively. In addition, Ostlund et al. reported significant improvements in the overall MCS and scores in the RE, VT, and SF subdomains after IVIg treatment, particularly in patients aged younger than 65 years and those with paresis of the lower limbs and high pain intensity (VAS score > 2 cm) [11].

### Cytokines

One RCT (compared with data from the placebo groups) [12] and 2 prospective trials (compared with pretreatment levels) [5,13] reported changes in the serum and CSF cytokine expression levels. The aforementioned RCT reported significantly decreased TNF-α levels in CSF cells (Table 3) [12]. In addition, the 2 prospective studies conducted by Gonzalez et al. reported significantly increased serum TNF-α levels and CSF TNF-α and IFN-γ levels in patients with PPS before IVIg treatment compared with those with other noninflammatory neurological diseases (ONDs) [5,13].

### Adverse effects of IVIg

Although Gonzalez et al. reported several adverse effects of IVIg treatment, it was well tolerated by most patients; no anaphylactic reactions or severe adverse events related to IVIg treatment were reported in the studies. The incidence of gastrointestinal disorders, general disorders, adverse reactions at the administration site, nervous system disorders, and skin and subcutaneous tissue disorders was higher in the active treatment group than in the control group, with headache being the most common side effect [10].

## Discussion

The present review showed that pain, a major outcome in all studies, improved after IVIg treatment, particularly in patients with PPS who exhibited high VAS scores before intervention, in younger patients, and in those with paresis of the lower limbs. However, when the overall data were subjected to meta-analysis, no significant differences were observed between the intervention and the placebo groups. The meta-analysis of the reduction of fatigue scores revealed no significant improvement after IVIg treatment. Regarding QoL, some prospective studies observed significant improvements in the overall PCS after IVIg treatment, but the RCTs revealed more controversial outcomes. Only scores in certain SF-36 subdomains significantly improved, particularly in patients aged older than 65 years and those with paresis of the lower limbs and high pain intensity. Moreover, only one RCT reported improvements in muscle strength and physical performance.

A study postulated that new-onset muscle weakness and decreased endurance of previously polio-affected muscles, cardinal symptoms of PPS, result from an uncompensated denervation–reinnervation process that leads to the loss of motor units [29]. All 3 RCTs and one prospective study explored the clinical relevance of IVIg in improving muscle strength by using a dynamometer; however, the results reported were inconclusive. Bertolasi [14], Farbu [12], and Kaponides [8] reported no obvious clinical amelioration of muscle strength after IVIg treatment. Gonzalez et al. [10] reported a median difference of 8.6% in favor of the intervention group. However, this result was not considered clinically relevant by the authors, as the target improvement at the start of their study was set at 15%. Moreover, the degree of decline in muscle strength in the placebo group was considerably higher than in previous reports on the natural course of untreated PPS patients. This might possibly be explained by differences in the study populations in these studies or more specific differences in the study muscles [3]. The discrepancies in these results might be due to the lack of uniformity in the study designs regarding the muscles selected and the methods used.

Regarding the cytokine expression levels, 2 studies reported that both serum and CSF IFN-γ and TNF-α levels were higher in patients with PPS than in the control group patients with ONDs. Although the OND patient cohort was heterogeneous, the patients with ONDs exhibited decreased cytokine expression levels in the blood and CSF [5]. Contrary to the healthy controls with no neurological disease, one study that evaluated serum inflammatory markers revealed significantly increased TNF-α, IL-6, and leptin levels in patients with PPS [30]. Moreover, several studies revealed evidence of inflammation in the muscles and the spinal cord in addition to an upregulated intrathecal synthesis of IL-4, IFN-γ, TNF-α, and IL-10 [31]. In addition, our review revealed that the CSF IFN-γ and TNF-α expression levels decreased remarkably within 1 to 2 months and even 1 year after IVIg treatment. Both IFN-γ and TNF-α are potent proinflammatory cytokines and are involved in several immunological processes. The findings of the present review support the potential pathogenesis of inflammation in PPS as well as the potential benefits of IVIg. However, the reason for the increased levels of cytokines in the CSF in PPS, occurring decades after acute poliovirus infection, remains unclear. Several hypotheses regarding the increased cytokine levels have been postulated: (1) poliovirus genomic particles potentially induce the production of cytokines, which then gradually contribute to chronic inflammation; (2) a poliovirus-related autoimmune response against unidentified neuronal or nonneuronal autoantigens; and (3) an immune response secondary to CNS damage that is not directly related to the symptoms [30,32]. One study revealed that the increase in TNF-α levels in PPS was associated with increased muscle pain but not with joint pain, muscle strength, fatigue, or disease duration [30]. However, none of the studies investigated the direct correlation between the symptoms and cytokine expression after IVIg treatment. Although lower CSF TNF-α shown in Farbu’s study and decrease IFN-γ shown in Gonzales’s study, both of which reported significant improvement of pain score, these were indirect finding. We cannot establish the relationship between the decrease of CSF TNF-α or IFN-γ and improvement of pain on the current evidence.

Furthermore, the studies used various intervention schemes. Gonzalez et al. administered a fixed IVIg dosage, which was repeated at 3-month intervals. By contrast, in Norwegian and Italian study groups, IVIg was administered according to the patients’ body weight during a single treatment period. In addition to PPS, numerous trials have investigated the clinical benefits of IVIg in immune-mediated diseases of the central and peripheral nervous systems, such as Guillain–Barré syndrome (GBS) and myasthenia gravis (MG). The EFNS guidelines recommend IVIg regimens of 0.4 g/kg/day for 5 days for GBS. For patients with MG, 2 g/kg of IVIg administered over 2 days was compared with 1 g/kg of IVIg administered in a single day, and a trend toward superior outcomes was observed at the higher dose [7].

Variability in the clinical factors and nonuniform reporting of the clinical parameters contributed to the heterogeneity among the reviewed studies. First, the characteristics of the participants varied considerably; for example, one study recruited 9 patients with weakness involving one or 2 limbs and 11 with more widespread weakness [12]. Second, the doses and duration of IVIg treatment varied among the studies. Third, the experience of the outcome assessors can affect the clinical outcomes. Fourth, the methods used for evaluating muscle strength and physical performance varied among the studies, indicating the possibility of measurement bias in our assessment.

Our review had several strengths; specifically, a comprehensive search for relevant studies was conducted, the eligibility criteria were applied systematically and explicitly, study quality was considered carefully, and a rigorous analytical approach was used. However, our review was limited by the methodological quality of the original studies (Table 2). First, several trials included small samples, and one study recruited only 10 patients in each treatment group [12], diminishing the statistical power of their analyses. Second, one study did not report adequate randomization in their study group allocation [14]. Third, only 3 RCTs were included, and their outcome assessments were evaluated using different methods; thus, the effect estimates of the efficacy of IVIg treatment could not be pooled. Furthermore, we had to estimate the mean and standard deviations for meta-analysis according to the reported median and interquartile range obtained from one RCT, potentially limiting the inferences based on our analysis [10].

## Conclusion

Although we observed statistically significant differences in the pain scores in each individual prospective trial, our meta-analysis of the RCTs indicated that the administration of IVIg treatment for PPS is unlikely to produce a significant reduction in the pain and fatigue severity, and improvement of muscle strength. Overall, the methodological quality of the reviewed studies was not adequate. Regarding the cost benefit, we cannot recommend the routine administration of IVIg for patients with PPS, but it could serve as a supportive treatment option for patient subgroups with moderate to severe PPS. Additional large, long-term RCTs are required to further evaluate the responding subgroups, long-term effects, and dosing schedules.
